# The MOMANT study, a caregiver support programme with activities at home for people with dementia: a study protocol of a randomised controlled trial

**DOI:** 10.1186/s12877-022-02930-x

**Published:** 2022-04-07

**Authors:** S. C. E. Balvert, M. V. Milders, J. E. Bosmans, M. W. Heymans, S. van Bommel, R.-M. Dröes, E. J. A. Scherder

**Affiliations:** 1 Department of Clinical Neuropsychology, Vrije Universiteit Amsterdam, van der Boechorststraat 7, 1081 BT Amsterdam, the Netherlands; 2 Department of Health Sciences, Vrije Universiteit Amsterdam, Amsterdam Public Health Research Institute, Amsterdam, the Netherlands; 3 Department of Epidemiology and Biostatistics, Amsterdam University Medical Centers, Location VU University Medical Center, Amsterdam, the Netherlands; 4 Management & Support, Stadionweg 53HS, 1077 RZ Amsterdam, the Netherlands; 5 Department of Psychiatry, Department of Research and Innovation, Amsterdam University Medical Centers, Location VU University Medical Center, GGZ inGeest, Amsterdam, the Netherlands

**Keywords:** Dementia, Informal caregiver, Psychosocial intervention, Randomized controlled trial, Activities at home

## Abstract

**Background:**

Because of the expected increase in the number of people with dementia, and the associated social and economic costs, there is an urgent need to develop effective and cost-effective care for people with dementia and their caregivers. The intervention proposed here combines two approaches to caregiver support that have shown to be effective in empowering caregivers, i.e., multiple components for caregiver support and actively engaging caregivers to involve the person with dementia in activities at home. The aim is to investigate whether the intervention is effective in improving quality of life in the caregiver and the person with dementia. A further aim will be to investigate whether this intervention can improve caregivers’ feeling of competence, experience of caregiving, and mood.

**Methods:**

The study design is a pragmatic, cluster randomised controlled trial with cost-effectiveness analysis. The study participants are informal caregivers and home-living persons with dementia for whom they care, recruited in various regions in the Netherlands. The trial will compare outcomes in two groups of participants: 85 dyads who receive the intervention, and 85 dyads who receive care as usual. The intervention is a caregiver support training that is manual based and consists of 6 group sessions over 2 months. Training takes place in small groups of caregivers led by a health care professional presented at dementia day care centres. Randomisation occurs at the level of the day care centre. Participants are assessed on the outcome measures at baseline, prior to the intervention, and at 3 and 6 months after baseline.

**Discussion:**

The study will provide insight into effectiveness and cost-effectiveness of an intervention that has not previously been evaluated or implemented in the Netherlands. The intervention potentially adds to the effective support options for informal caregivers of people with dementia without greatly increasing the workload for health- or social care professionals.

**Trial registration:**

The trial is registered at the Dutch Trial Register at NTR6643; August 22^nd^, 2017.

## Background

Currently, it is estimated that there are 50 million people living with dementia worldwide, and this number is expected to increase to approximately 150 million by 2050 (Organization [32]). The majority of people with dementia are cared for by their family members or other informal caregivers at home in the community. As a consequence, they are less likely to be hospitalised or move into residential care and tend to have a better quality of life than those in care homes (Charlesworth, Burnell et al. [5]). However, compared to other caregivers, the informal caregivers of people with dementia experience a greater burden and distress (Ory, Hoffman III et al. [34]). Caring for a person with dementia is described as a chronic stress experience, and caregivers report feeling isolated and overburdened (Szcześniak, Rymaszewska et al. [43]). In the Netherlands, more than half of the caregivers of people with dementia have reported to be quite heavily overburdened, and almost half of the caregivers also reported to be the only one responsible for the care of their loved one or parent (Heide, Veer et al. [19]). The prevalence and incidence of depression and anxiety disorders is high among informal caregivers of older adults, whereas they show less preventive and self-care behaviours, more impairments in physical health, greater acute care utilization, and even increased mortality (Stall, Kim et al. [42]). Van ‘t Leven et al. [50] found that the functioning of the person with dementia was directly related to the functioning of the informal caregiver. As such there is an urgent need to develop interventions for people with dementia and carers that are supportive for both and will improve their quality of life. Multi-component psychosocial interventions combine therapeutic strategies directed at the informal caregiver or the person with dementia or both, and appear particularly effective in improving caregiver well-being, as well as maintaining well-being and daily functioning in the person with dementia (Olazarán, Reisberg et al. [29], Chien, Chu et al. [6], Milders, Bell et al. [25], Van’t Leven, de Lange et al. [50], Sikkes, Tang et al. [39])**.**

Caring for people with dementia living at home is challenging, which is why home-based psychosocial interventions are highly needed to reduce caregiver burden. There is preliminary evidence to support the acceptability of psychosocial interventions for dementia caregivers (Qiu, Hu et al. [35]). For example, it has been found that peer support can significantly improve well-being of caregivers by decreasing feelings of isolation, encouraging better coping strategies, and enabling a change in caregiving behaviour (Brodaty and Donkin [4]), Charlesworth, Burnell et al. [5], (Organization [31]), Sousa, Sequeira et al. [40]). Generally, caregivers often lack social contact and support and experience feelings of social isolation (Brodaty and Donkin [4]), Szcześniak, Rymaszewska et al.[43]), because they lack personal time and opportunities to socialise, and also experience stigma resulting in family and friends distancing themselves (Charlesworth, Burnell et al. [5], Dröes, Meiland et al. [12]). Interventions can offer support to caregivers of people with dementia and can reduce the pressure and distress for the caregiver, which can be of benefit to both the caregiver as well as the person with dementia. Moreover, if nursing home admission of the person with dementia can be postponed (Moon and Adams [27]), the pressure on health and social care services can be reduced (Stall, Kim et al. [42]). Additionally, the costs for long-term residential care of people with dementia could also be reduced, which is important because these are especially high in the Netherlands, and even among the highest in the European Union (Wimo, Jönsson et al. [56]). However, to date there are few effective interventions for caregivers of people with dementia that are widely available. In a review by Sousa et al. (Sousa [41])it was found that the main characteristics of interventions that successfully addressed caregivers’ needs were: (i) six weekly sessions with an average duration of 100 min,(ii) preferred topics ranged from learning and training skills in physical and mental health issues to knowing where to find and how to use available health services; and (iii) greater family and peer support. Based on interviews with caregivers of persons with dementia in different stages of the disease, Boots et al. (Boots [2]) recommended that therapeutic interventions should not be problem-oriented, but instead (i) try to identify the needs of the caregivers’ individual situation, (ii) focus on adaptation rather than on loss, and (iii) increase their knowledge of the disease and future changing roles to reduce the negative consequences of caregiving later on. In turn, caregivers found that the available information on dementia was negative and stigmatizing, and caregivers of persons in more advanced stages of dementia thought that disease-specific knowledge was crucial to help them understand the person with dementia, and improved their ability to empathize with their loved one (Boots, Wolfs et al. [2]).

Although multi-component psychosocial interventions seem to be effective, few attempts have been made to implement these interventions into practice (Gitlin, Marx et al. [14]): translation from randomized trial to implementation into social and support services still seems to be complicated, and large local differences between availability and accessibility of these health care services seem to exist (Birkenhäger-Gillesse, Kollen et al. [1]). A reason for this limited implementation and availability could be the high costs of most current multi-component interventions, and the fact that the interventions rely heavily on already overburdened health care professionals: interventions require time for health care professionals to learn how to present the intervention, but also require time to actually deliver the intervention to caregivers or people with dementia and can in such a way be very labour intensive in practice (Graff, Vernooij-Dassen et al. [16], Milders, Bell et al. [25]). In a recent study by Christie (Christie [7]), it was even found that the implementation of caregiver support programs was often the sole responsibility of a single person within the municipality instead of a team that could tackle implementation issues together. Interventions seemed to have low priority within organisations, even though these were fitting to caregivers’ needs, and incentives or rewards to encourage implementation into clinical practice were often absent (Christie [7]). The authors concluded that implementation needed more engagement of local health care organizations, and that reflecting on and evaluating the implemented intervention should have more priority in the future (Christie [7]).

Therefore, in the current study, a caregiver support program to promote activities at home for people with dementia will be adapted for use and implemented in community based social service settings in the Dutch health care system and evaluated on its effectiveness and cost-effectiveness. To reduce staff costs and demand on health services, this study involved informal caregivers of people with dementia to present part of the intervention and recruited staff from local health care and welfare organizations to train the caregivers. The intervention was manual based to limit the time required for staff to prepare the sessions, and also increase information availability for the caregivers and promote treatment fidelity and compliance with the intervention. The intervention in this study, the so called MOMANT caregiver support program, combines two approaches to caregiver support: first, it is a multi-component intervention aimed at both the person with dementia and the caregiver, using evidence-based components of caregiver support, such as education, peer support, and activity engagement. Previous studies have shown that training family caregivers to present interventions is feasible and acceptable for both people with dementia and caregivers (Teri, Logsdon et al. [46], Quayhagen [36], Gitlin, Winter et al. [15], Milders, Bell et al. [26], Milders, Bell et al. [25]). Trials of combined multicomponent interventions have shown their effectiveness at improving caregiver well-being and postponing institutionalization of the person with dementia with several months to a year (Dröes, Breebaart et al. [11], Olazarán, Reisberg et al. [29], Chien, Chu et al. [6], Van't Leven, Prick et al. [49]). Second, to promote effective implementation of the intervention the informal caregivers are trained to engage the person with dementia in stimulating activities. Previous research showed that this approach can have positive effects on the wellbeing of the caregiver and the person with dementia, as well as on the cognitive functioning of the person with dementia, and it can even improve sense of competence of the caregiver (Teri, Gibbons et al. [44], Onder, Zanetti et al. [30], Graff, Vernooij-Dassen et al. [16], Orgeta, Leung et al. [33]).

### Aim

The main aim of the study is to investigate whether an intervention to educate and train caregivers and to empower caregivers to engage the person with dementia in stimulating activities at home, is effective and cost-effective to improve quality of life in the caregiver and the person with dementia in comparison with usual care; and as such the intervention might be able to contribute to the availability and accessibility of health care services. A further aim will be to investigate whether this intervention can improve caregivers’ feeling of competence, experience of caregiving, and mood.

## Methods

### Design

The study design is a cluster randomized controlled trial. The trial will compare outcomes in two groups of participants: 1) caregivers who receive the intervention and the person with dementia for whom they care; 2) caregivers and the person with dementia for whom they care, who continue to receive usual care. The intervention will be implemented at community day care centres, support centres- and Meeting Centres for people with dementia and carers (hereinafter summarized as day care centres). Randomisation will occur at the level of the centre. Participants recruited at day care centres allocated to the intervention condition will receive the MOMANT caregiver support program, and participants recruited at day care centres allocated to the control condition will continue to receive usual care. Data are collected at baseline, and at 3 and 6 months after baseline.

### Setting

The MOMANT project (registered at the Dutch Trial Register; Trial ID, NTR6643) will be coordinated at the Vrije Universiteit Amsterdam and will involve organisations for care and welfare of the elderly from across the Netherlands. Recruitment of participants, and presentation of the intervention in the intervention condition, will take place at the day care centres by staff of the organisation.

### Participants

The study participants will be dyads consisting of an informal caregiver and the person with dementia for whom they care.

#### Eligibility criteria

Informal caregivers can be spouses, relatives, or friends who care for and support the person with dementia without receiving payment for performing care. If the caregiver does not live with the person with dementia, they have to visit the person with dementia at least 3 times a week to be eligible for the study.

The person with dementia is living at home, cared for by the caregiver, and should have a formal diagnosis of dementia or experience severe cognitive impairments influencing their daily functioning such that dementia is strongly suspected. Type of dementia is not an inclusion criterion. The person with dementia did not start with dementia-specific medication less than 6 months before inclusion.

Exclusion criteria for both the caregiver and the person with dementia will be major mental or physical illness, such as major depression or stroke, that would affect their ability to participate in the training, the intervention or complete the assessments. A second exclusion criteria will be participation in another intervention study.

### Recruitment

Participants will primarily be recruited through health care professionals of day care centres, who are already in contact with the target population. Contact will be made through case managers and day-care coordinators, who will notify potential participants about the study and distribute written information about what participation entails. Contact details of potential participants are only passed to the research team with permission of the potential participants.

### Randomisation

Randomisation occurs at the level of the day care centre. The allocation schedule is produced by a computer-generated random sequence. All dyads recruited at a centre will enrol in the condition to which the centre has been allocated.

### Sample size

The sample size estimation is based on the primary outcome measures of the caregivers and the persons with dementia. At least 64 dyads in each group are required to achieve 80% power to detect significant differences between groups of half a standard deviation on the primary outcome measures using independent sample analyses with an alpha level of 0.05 (Cohen [9]). This estimate assumes a medium effect size (Cohen’s d = 0.5) on the main outcome measures, which is based on effect sizes from comparable interventions in caregivers with an average effect size of d = 0.41 (Olazarán, Reisberg et al. [29]). With an anticipated drop-out rate of 25%, 85 dyads would need to be recruited in each group to end up with 64 dyads completing the study, which is why the aim is to recruit 170 dyads in total.

### Intervention

Caregivers in the intervention condition will receive the MOMANT manual-based training and support program consisting of multiple components, including special attention for the implementation of activities at home: 1) educating caregivers on effective communication with the person with dementia; 2) teaching skills of how to manage difficult behaviour of the person with dementia; 3) suggesting methods of how to cope with the burden of caregiving; 4) providing detailed suggestions of how to engage the person with dementia in activities that are enjoyable and stimulating, physically, cognitively or socially in a person-centred way. That is, the activities should match the interests and abilities of the person with dementia and the caregiver and may include activities such as household tasks, going over news headlines, reminiscence activities, walking or other simple physical activities. As such, the manual consists of an overview of these topics, details of activities and examples of materials to be used in activities, and additional information, e.g., useful website links and telephone numbers on where to find and how to use available health services. Education and training of caregivers will take place in small groups (4–8) at the day care centres, led by a health care professional, during 6 sessions over 2 months. Each session will last approximately 1 h. The first 3 sessions will be held weekly, the subsequent 3 sessions will be held with intervals of two weeks.

Although the precise content of the sessions will in part be determined by caregivers’ needs and wishes, the program consists of the following sessions:

-
  1. Introduction and explanation rationale and goal of the training, education on different types of dementia and the consequences, and general suggestions for a healthy lifestyle
  2. Stress of caregiving and coping strategies to reduce behavioural disruptions in people with dementia, and practicing communication techniques
  3. How to present the activities, basic principles and how to motivate the person with dementia
  4. Implementation of activities at home
  5. Caregivers’ experience of presenting activities at home, discuss difficulties and possible solutions
  6. Caregivers’ overall experience with the intervention, revisit topics presented in earlier sessions

From meeting 3 onwards, caregivers will be encouraged to start with activities at home with the person with dementia. During the remaining sessions, time will be reserved for feedback from caregivers and further support on how to implement activities at home.

The caregiver training will take place at day care centres for people with dementia and their caregivers. The participating caregivers will attend a day care centre in their area to join the training, which provides the opportunity that the person with dementia is looked after if needed, while their caregiver is attending the training.

To facilitate implementation of the intervention at home and to assess intervention fidelity, the health care professional who provides the training will also visit the dyad once at home. This visit will be scheduled to provide the caregiver with an individual session to ask questions as opposed to the group sessions and to allow the professional to see how the activities are performed at home. The health care professional will advise on how to implement the activities at home over a longer term. After the visit the professional will rate how well the caregiver presents the activities at home as part of the assessment of intervention fidelity.

Health care professionals involved in the study will themselves be trained by the research team on how to offer the intervention. As these professionals already have extensive experience in dementia care, this training of the trainers will take place in a single session of 2 to 2.5 h. In this session, trainers will also receive a copy of the manual for caregivers, which is the basis for the caregiver training, and a trainer’s manual which outlines the content of the intervention sessions.

The manual for caregivers and trainers will be adopted from material developed earlier (Milders, Bell et al. [26], Milders, Bell et al. [25]). Finalising the manual content will also occur in consultation with caregivers, people with dementia, and professionals in dementia care as part of the focus group that will be set up at the start of the study.

### Treatment as usual

Participants in the control condition will continue to receive treatment and care as usual. Common practice regarding dementia care in the Netherlands varies between regions, but most community-dwelling persons with dementia are cared for by their spouse or relative in their own home, and attend day care facilities outside their home one or more days per week. Participants randomised to the control group can continue with any health, social or voluntary sector services they are currently receiving or commence once recruited into the study. Participants who are allocated to the control condition will also be offered the opportunity to receive the intervention material after completion of the final assessment.

### Measures

Data from participants in the intervention and control conditions will be collected by trained researchers on three occasions, i.e., at baseline, prior to the start of the intervention; 3 months after baseline; and 6 months after baseline. Nursing home admission will be recorded until shortly before the study ends by means of a telephone conversation with the caregiver 12–18 months after the final assessment.

The primary outcome is health-related quality of life for the caregiver. Secondary outcomes for the caregiver include the caregivers’ feeling of competence to care for the person with dementia, caregivers’ experience of caregiving, and mood. The primary outcome for the person with dementia is self-reported quality of life. Secondary outcomes for the person with dementia include activities of daily living, global functioning, and the frequency of activities enjoyed by the person with dementia; as reported by the caregiver. Participants are also asked about health resource use and caregiver time inputs to explore cost-effectiveness.

### Primary outcome

The primary outcome measure for the caregiver will be health-related quality-of-life assessed with the self-reported EQ-5D-5L instrument, including visual analogue scale (VAS) (Herdman, Gudex et al. [20], Versteegh, Vermeulen et al. [52]). The descriptive system comprises five dimensions: mobility, self-care, usual activities, pain/discomfort and anxiety/depression, scored on a five-point scale. A higher score corresponds to more severe complaints, and thus a lower quality of life. The EQ-5D-5L has proven to be a valid and reliable questionnaire to measure quality of life in several populations (Janssen, Birnie et al. [22], Feng, Kohlmann et al. [13]), and the VAS has shown good convergent validity (Tran, Ohinmaa et al. [48]).

The primary outcome measure for the person with dementia will be quality of life assessed with the self-reported Dementia Quality of Life scale (DQOL; (Brod, Stewart et al. [3]) comprising of 29 items, ranked on a five-point Likert scale and measuring five QoL domains: self-esteem, positive affect, negative affect, feeling of belonging and sense of aesthetics. A higher score on the DQOL means a better quality of life, except on the negative affect outcome. The questionnaire has shown good internal consistency, and good convergent validity (Wolak-Thierry, Novella et al. [57]). The DQOL is an appropriate research tool for people with dementia, because it has been used extensively in this population, and has proven good discriminant validity (Wolak, Jolly et al. [58], Wolak-Thierry, Novella et al. [57]).

### Secondary outcomes

The Resource Utilization in Dementia (RUD; Wimo, Gustavsson et al. [55]) will be used to collect socio-demographic characteristics of the caregivers and persons with dementia including age; gender; relationship with person with dementia; employment status; and living situation. Furthermore, additional questions regarding the in- and exclusion criteria will be asked during the first visit: dementia diagnosis related information, whether major events occurred in the last 3 months that might affect well-being, whether they have mental or physical problems that might hinder participation, and whether they receive any other support or training through another intervention of the day care centre.

Sense of competence: The Short Sense of Competence Questionnaire (SSCQ; Vernooij‐Dassen, Felling et al. [51]) is a seven-item questionnaire that specifically measures a caregiver’s satisfaction and worries in their role as caregiver. In this shorter version the items refer to three domains: the satisfaction of the caregiver with the person with dementia as recipient of care; the satisfaction of the caregiver with their own performance as caregiver; and the negative consequences that caring has for the social and personal life of the caregiver. The three subscales showed good homogeneity and feasibility, also when comparing the population of informal caregivers of patients with diagnosed dementia with the caregivers of older adults with dementia symptoms (Jansen, van Hout et al. [21]). A higher score indicates less sense of competence in dealing with the burden of caregiving.

Positive experiences through informal care: Positive Experiences Scale (PES; De Boer, Oudijk et al. [10]) consist of eight items to measure positive experiences by informal caregivers. It varies from intrinsic satisfaction and relational enhancement to improvement of competence and social enhancement. For caregivers of persons with dementia, the authors of the PES recommend to exclude two items (‘because of the caregiving the relation with my family and friends has become closer’ and ‘I receive a lot of appreciation for the care I gave’), because these do not apply to informal caregiving of dementia. The total score is calculated by adding up all individual scores, where a higher score indicates a more positive experience towards caregiving. The PES had good reliability (α = 0.74) and is recommended because of its psychometric qualities and its usefulness in different populations of informal caregivers (De Boer, Oudijk et al. [10]).

Mood: The Center for Epidemiologic Studies – Depression (CES-D1; Radloff (Radloff [37])is a brief self-report scale designed to measure self-reported symptoms associated with depression experienced in the past week. It consists of 20 items comprising six scales reflecting major facets of depression: depressed mood, feelings of guilt and worthlessness, feelings of helplessness and hopelessness, psychomotor retardation, loss of appetite, and sleep disturbance. A higher score means the presence of more depressive symptoms. The CES-D1 does not measure chronic depression, but current levels of depressive symptomatology, and demonstrated strong internal consistency with Cronbach’s α ranging from 0.79 to 0.90 in caregiving and other medical studies (Clark and Diamond [8]).

Use of health care: An adapted version of the RUD (Wimo, Gustavsson et al. [55]) will be used to measure healthcare resource utilization among persons with dementia and their caregivers, and time spent on formal and informal care by caregivers. The RUD is a widely used instrument to assess resource use in dementia. The RUD will be completed by the caregiver’s report on healthcare resource utilization of themselves and of the person with dementia.

Pleasant events: the Pleasant Events Schedule-Alzheimer’s Disease (PES-AD; Teri and Logsdon (Teri and Logsdon [45]) Short Form is a commonly used self-report assessment that attempts to identify activities that individuals with dementia may find enjoyable. It is used to assess the overall frequency of engagement in 20 activities by the person with dementia, as reported by the caregiver. A higher score indicates that the person with dementia is engaged in and enjoyed more activities. The PES-AD had good predictive validity, and most participants endorsed the majority of items indicating that this measure accurately identified items that are likely to be enjoyable by older adults (LeBlanc, Raetz et al. [24]).

Activities of daily living: Interview for Deterioration in Daily Living Activities in Dementia (IDDD; Teunisse and Derix (Teunisse and Derix [47]), a caregiver-based measure which consists of 33 items, reflecting the initiative to perform and actual performance of self-care and more complex activities, as reported by the caregiver. A higher score indicates more cognitive impairment in daily life. The IDDD has good construct validity and test–retest reliability (Voigt-Radloff, Leonhart et al. [53]).

Global functioning: Global Deterioration Scale (GDS; Reisberg, Ferris et al. [38]) denoting presence and severity of dementia. The GDS identifies seven stages of severity (1 least severe – 7 most severe) based on the estimated amount of cognitive decline. The GDS is completed in a structured interview with the caregiver. The GDS has been reported to have good interrater reliability, ranging from 0.82 – 0.92 (Wesson and Luchins [54]).

Time of admission to residential care, as reported by caregiver.

### Process evaluation

In addition to the formal outcome measures, further data will be collected to allow a process evaluation to identify possible facilitators and barriers to implementation of the intervention, thus potentially influencing its effectiveness. For this purpose, caregivers’ attendance at the training sessions will be recorded and caregivers in the intervention condition will be invited to complete a short questionnaire to evaluate the intervention at 3-month and at 6-month follow-up. At the 3-month follow-up, caregivers will be asked about the activities they do at home with the person with dementia (e.g., frequency, type of activities), difficulties implementing suggestions and recommendations from the training at home and whether the person with dementia and they themselves enjoyed the activities. At the 6-month follow-up, caregivers will be asked similar questions as well as whether they still engage in the activities at home. Health care professionals who presented the intervention will be asked to evaluate the training that they have received from the research team and the strengths and weaknesses of the intervention that they encountered while training the caregivers. All evaluation questionnaires can be returned anonymously to the researchers, to encourage honest responding and prevent socially desirable responding. For process evaluation, the British Medical Research Council guidance will be used as framework (Moore, Audrey et al. [28]).

### Intervention fidelity

To assess whether the health care professionals presented the caregiver training as intended, a sample of sessions from the caregiver training will be video-recorded, with permission from the caregivers and trainers. The recording will be scored by two independent raters from the research team against a list of predetermined criteria. To assess whether the informal caregivers follow the instructions from the training at home and carry out activities with the person with dementia as intended, the activities presented at home will be scored during a home visit by the health care professional who provides the training sessions, with permission of the dyad.

### Blinding

This study is single blinded. It is not possible to blind participants or health care professionals to their allocated condition in this psychosocial intervention. However, the researchers who administer the outcome measures will be blinded to the group allocation to try to minimize the information bias effect. To reduce the risk that dyads disclose their group during the assessments, dyads are asked not to inform the researcher of the intervention until the end of the assessment. In order to still be able to ask questions about the participants’ experiences with the intervention at the 3- and 6-month follow up measurements, the researcher will be allowed to ask the condition of the participant at the end of the assessment session. In order to maintain researchers’ blinding of group allocation, the same researcher will not perform the next follow up measurement of the same dyad.

### Ethical arrangements

This study is conducted according to the principles of the Declaration of Helsinki and all participants will give informed consent. This study has been considered by the Medical Ethics Committee of the VU University Medical Centre in Amsterdam not to fall under the Medical Research Involving Human Subjects Act. The ethics committee of the Faculty of Behavioural and Movement Sciences of the Vrije Universiteit Amsterdam has approved the study (VCWE-2017–015).

#### Consent

The nature of the research will be explained to all potential participants orally and by written information, after which dyads will have two weeks to decide whether to participate. All those who decide to take part in the study will sign an informed consent form. If people with dementia are not able to sign due to vision or writing impairments, their informal caregiver will provide consent on behalf of the person with dementia.

### Data management / statistical analysis

Data collected from participants will be stored pseudonymized, personal information will be removed and participants will identified by a number. A separate restricted-access key file will link the number with the personal information. Following completion of the study and data verification the key file will be deleted and data will be anonymised.

Data will be analysed according to the intention-to-treat principle using IBM SPSS Statistics 24 (Version 24.0; IBM Corp., 2016). All participants who entered the study will be included in the analysis according to the condition in which they had initially started. To analyse the primary outcome measures, repeated measures regression analyses (linear mixed models) will be used in order to handle clustered data and to maximise the statistical power comparing the intervention and control group at baseline, 3 and 6 months follow up. The differences between the intervention and control group at each follow-up measurement will be obtained from the linear mixed models by including time as a categorical variable and the interaction between time and the intervention group variable. Secondary analyses will examine post intervention group differences in caregivers’ sense of competence, experience of caregiving, mood, and activities enjoyed by the person with dementia using regression analyses, including baseline data as a covariate.

Analyses will be adjusted for baseline scores and potential confounding variables, i.e., factors that differ between the groups at baseline and are related to the outcome measures (at baseline) and thus might influence the impact of the intervention, e.g., demographic variables, by introducing these factors into the regression models. Baseline differences in demographic and clinical characteristics will be investigated on the cluster and individual participant level using Chi-square tests, Mann Whitney U tests, *t*-tests, and analysis of variance (ANOVA) depending on the type of variables (nominal, ordinal, interval variables).

The data collected for the process evaluation and intervention fidelity will be analysed using descriptive statistics. The data on intervention fidelity will also be used as a classification factor in the effect analysis to identify whether this makes a difference in the effect outcomes. All tests will be carried out with a 5% significance level.

### Cost-effectiveness analysis

The aim of the economic evaluation is to relate the difference in societal and healthcare costs between the intervention and the control condition to the difference in effect on the outcome measures. Both a cost-effectiveness and cost-utility analysis will be performed with a time horizon of 6 months.

#### Cost analysis

Healthcare costs include costs of primary and secondary care, complementary care, and home care. Other costs include costs of informal care, meal services, and transportation services. Societal costs comprise of both healthcare and other costs. For the valuation of the health care utilization, standard prices published in the Dutch costing guidelines will be used (Hakkaart-van Roijen, Van der Linden et al. [18]). Medication use will be valued according to prices of the Royal Dutch Society for Pharmacy (Z-index. G-Standaard. The Hague, The Netherlands: Z-Index, 2002). Lost productivity of the caregiver will be assessed using the Productivity and Disease Questionnaire (PRODISQ; Koopmanschap (Koopmanschap [23]). The friction cost approach will be used to estimate lost productivity costs.

#### Patient outcome analysis

Societal costs will be related to the following effect measures in the economic evaluation:

(i) quality-adjusted life-years (QALYs) based on the Dutch tariff for the EuroQol (EQ-5D-5L; Group [17]) at the level of the caregiver; (ii) quality of life of the person with dementia using the Dementia Quality of Life scale (DQoL). The analysis will be performed according to the intention-to-treat principle. Missing cost and effect data will be imputed using multiple imputation. Incremental cost-effectiveness ratios (ICERs) will be calculated by dividing the difference in mean total costs between the treatment groups by the difference in mean effects. Bootstrapping with 5000 replications will be used to estimate the 95% confidence intervals around cost differences and the uncertainty surrounding the ICERs. Uncertainty surrounding the ICERs will be graphically presented on cost-effectiveness planes. Cost-effectiveness acceptability curves showing the probability that the intervention is cost-effective in comparison with usual care for a range of different ceiling ratios will also be estimated.

## Discussion

Because of the expected increase in the number of people with dementia worldwide, there is an urgent need to develop, evaluate and effectively implement evidence-based psychosocial interventions to support informal caregivers of people with dementia. If the caregiver feels better equipped to care for the person with dementia, a better quality of life can be maintained by both the caregiver and the person with dementia, and placement in a nursing home can be delayed. This paper describes the study protocol of a randomised controlled trial of the MOMANT caregiver support program for caregivers of home-dwelling people with dementia. This intervention, which combines multiple proven effective support components and pays much attention to its implementation, gives caregivers the tools and techniques to better care for their relative with dementia, but also engages the person with dementia through participation in stimulating activities. In addition to the RCT, a process evaluation is carried out to identify possible facilitators and barriers to implementation of the intervention as well as intervention fidelity, which may potentially influence its effectiveness. We anticipate that all data collection will be completed by December 2021.

A major strength of the current study is that the MOMANT program will take place at local health care organizations in such a way that staff costs and demand on health care services are reduced by training the informal caregiver to provide part of the intervention, and by working with an implementation manual. If study outcomes confirm the effectiveness of the implementation of this intervention, the MOMANT caregiver support program can be further implemented in real-world practices and routine care pathways, thus increasing the number of effectively supported informal caregivers. A second strength of this study is that the intervention includes activities for the caregivers and persons with dementia to do together in order for the person with dementia to be able to participate again in day-to-day life. Additionally, the fact that participants are included across different parts of the Netherlands ensures diversity in the group such that contextual differences can also be assessed. However, letting informal caregivers present part of the intervention could also be considered a limitation in terms of intervention fidelity, resulting in less visibility in the actual practice of how the intervention is presented. Moreover, because of the cluster randomisation there could be a risk of between-group differences at baseline, as well as only recruiting people with dementia who already receive professional (day)care. Despite these limitations, evaluating the effectiveness of this intervention will be a starting point in offering more support to this group of people in the routine care pathways, and therefore also be beneficial for those who do not yet receive professional care.

Effective psychosocial interventions for community-dwelling people with dementia and their caregivers are of the utmost relevance because the number of people suffering from dementia will continue to rise in the years to come, and the large majority lives and are cared for at home by informal caregivers. This study will not only contribute to the knowledge on the effectiveness of a multicomponent training and support intervention, but also on its implementation in regular care. Through this intervention, we hope to empower both the caregiver and the person with dementia to live a good life with dementia and to become more capable in dealing with future challenges.

## Data Availability

All research data will be archived and securely stored for 10 years after the end point of the study. The datasets generated during and/or analysed during the current study are available from the corresponding author on reasonable request.

## Abbreviations

CES-D1: Center for Epidemiologic Studies – Depression
DQOL: Dementia Quality of Life
GDS: Global Deterioration Scale
ICERS: Incremental cost-effectiveness ratios
IDDD: Interview for Deterioration in Daily Living Activities in Dementia
PES: Positive Experiences Scale
PES-AD: Pleasant Events Schedule-Alzheimer’s Disease
PRODISQ: Productivity and Disease Questionnaire
QALY: Quality-adjusted life-years
RCT: Randomized controlled trial
RUD: Resource Utilization in Dementia
SSCQ: Short Sense of Competence Questionnaire
VAS: Visual Analogue Scale
VU: Vrije Universiteit
WHO: World Health Organisation
